# Inconsistent seduction: Addressing confounds and methodological issues in the study of the seductive detail effect

**DOI:** 10.1002/brb3.2322

**Published:** 2021-08-22

**Authors:** Kay L. Tislar, Kelly S. Steelman

**Affiliations:** ^1^ Department of Cognitive and Learning Sciences Michigan Technological University Houghton Michigan USA

**Keywords:** instructional design, learning objectives, methods, multimedia learning, seductive detail effect

## Abstract

**Introduction:**

The inclusion of interesting but irrelevant details in instructional materials may interfere with recall and application of the core content. Although this seductive detail effect is well researched, recent research highlights factors that may influence the effect size.

**Objectives:**

The current study discusses confounds and methodological issues in the study of seductive details and outlines strategies for overcoming them. These practices were then applied in a study that examined the role of learning objectives on the seductive detail effect.

**Methods:**

Seductive details were selected on the basis of interest and importance level and matched for word count and reading level. The 3 × 2 between‐subjects design presented 132 undergraduate students with a lesson on plate tectonics; participants completed tests on both recall and transfer.

**Results:**

Results did not reveal a consistent detrimental effect of high‐interest details on core content recall and transfer. On the recall test, contrary to expectation, the seductive detail effect obtained only when objectives were provided. A similar pattern emerged on the transfer task.

**Conclusion:**

These findings highlight the difficulty of consistently eliciting the seductive detail effect. We discuss outstanding issues that must be addressed in order to develop practical guidelines on the inclusion of seductive details in educational materials.

## Graphical abstract

The current study discusses confounds and methodological issues in the study of seductive details and outlines strategies for overcoming them. These practices were then applied in a study that examined the role of learning objectives on the seductive detail effect, using details selected on the basis of interest and importance level and matched for word count and reading level. Results did not reveal a consistent detrimental effect of high‐interest details on core content recall and transfer.

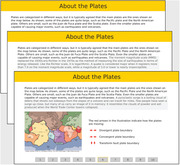

## 1 INTRODUCTION

Educators have long struggled with how to engage learners who may not find lesson content inherently interesting. In an attempt to capture and hold learners’ attention, some educators enhance possibly not‐so‐interesting educational materials with spiced‐up details, jokes, cartoons, fun facts, videos, animations, and songs—even if the information is not directly relevant to the instructional objectives. These types of enhancements are commonly referred to as *seductive details*, “interesting but irrelevant details that are added to a passage to make it more interesting” (Harp & Mayer, 1997). Typically, these details contain information that is tangential to the main ideas of a lesson, but that may be memorable because it is related to newsworthy or even lurid topics, including death, celebrities, and sex (Lehman et al., 2007). Although the intention is to keep learners engaged with the core material, Mayer (2005) has posited a *seductive detail effect*, which holds that people learn more deeply from material that does not include seductive details and that such details may even impede learning.

The purpose of the current study was to address confounds and methodological issues that have been raised regarding some seductive detail studies to determine if the effect manifests when some of these issues are addressed.

### 1.1 Theoretical foundations

Cognitive load theory (CLT), developed in the 1980s, is one of the main theories that has been used to help apply our knowledge of cognitive structures to instructional design (Sweller, 1988). The architecture upon which CLT is based centers on a limited‐capacity working memory system. CLT suggests that learners can absorb and learn information only if it is presented in a way that does not overload working memory. Instructional designers must, therefore, be mindful of learners’ cognitive load, defined as the total amount of effort imposed on working memory at a given time by the information being presented (Paas & Sweller, 2014).

Over the past 25 years, Richard Mayer and colleagues (Mayer, 2014) have investigated many of the issues related to the effects of instructional materials on cognitive load. Mayer developed a cognitive theory of multimedia learning (CTML), centered on the principle that learners attempt to build meaningful connections between words and pictures and learn more deeply from words and pictures than from words or pictures alone (Mayer, 2014). According to CTML, one of the principal aims of multimedia instruction is to encourage the learner to build a coherent mental representation, or schema, from the presented material. The learner's job is to make sense of the presented material as an active participant, ultimately constructing new knowledge.

**FIGURE 1:**
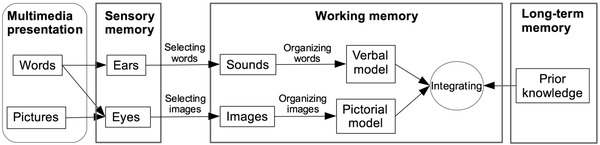
Mayer's cognitive theory of multimedia learning. Boxes represent memory, and arrows represent cognitive processes. (Stanislaus Erhardt, 2013, via Wikimedia Commons. Used and adapted under Creative Commons Attribute 3.0 License.)

Figure 1 provides an overview of how information is processed according to CTML. The illustration shows that two separate, but connected, subsystems are used for processing visual and auditory information, as in CLT. When we see or hear information, it initially passes through sensory memory. Because the sensory memory channels have limited capacity, we are unable to take in all of the information to which we are exposed; we must select the words or images that we find relevant and store those in working memory as mental representations of the actual sounds and images. Next, we organize the words and images by making connections between them to develop coherent models. Finally, we integrate the verbal and pictorial models with prior knowledge that we have stored in long‐term memory.

The CTML is based on three cognitive science principles of learning: the dual channel assumption, the limited capacity assumption, and the active processing assumption (Mayer & Moreno, 1998, 2003). The *dual‐channel assumption* contends that working memory has separate, but interconnected, auditory/verbal and visual/pictorial channels. It is based on Baddeley's (1974) theory of working memory and Paivio's (1986; Clark & Paivio, 1991) dual‐coding theory. Paivio's (1986) theory assumes that we have separate systems for processing verbal (words) and nonverbal (pictures, smells, and sounds) information as discussed above.

The *limited capacity assumption* is based on cognitive load theory (Sweller, 1988, 1994) and states that each of the two working memory channels can process a limited amount of information at one time.

The *active processing assumption* suggests that “people actively engage in cognitive processing in order to construct a coherent mental representation of their experiences” (Mayer, 2014, p. 50). Active learning requires three main cognitive processes: selecting relevant words and images for transfer to working memory, mentally organizing the selected words and images into a coherent model in working memory, and integrating the models with each other and with relevant knowledge from long‐term memory. Active processing is required for learning to occur, and much of this cognitive processing takes place in working memory.

The task of instructional designers is to create situations in which learners have enough resources to organize information into a coherent mental model and integrate it with prior knowledge, without overloading learners’ working memory capacity. Similar to CLT, CLTM defines different types of demands on a learner's information processing system that designers should consider when developing multimedia resources, one of which is extraneous processing.

Extraneous processing is processing that does not support the instructional goal and is caused by poor instructional design. One of the instructional design goals under CTML is to establish effective techniques to reduce extraneous processing (Mayer, 2014, p. 63). The challenge for instructional designers is to avoid extraneous overload, which occurs when cognitive processing exceeds a learner's cognitive capacity (Mayer & Fiorella, 2014). This type of overload can occur when either the visual or verbal processing channel—or both—is overloaded. It can occur when materials contain “too much detail, embellishment, or gratuitous information or when the layout of material is confusing” (Mayer & Fiorella, 2014, p. 281).

CTML has yielded several theory‐based instructional design principles designed to reduce extraneous overload. One of these is the coherence principle, which states that people learn more deeply from multimedia when extraneous material is excluded (Mayer, 1999). Employing the coherence principle enables instructional designers to eliminate interesting but irrelevant information—seductive details—in their materials so that learners have more cognitive capacity available for essential (intrinsic) processing, which is needed to comprehend the material and to represent the material in working memory.

### 1.2 Seductive detail paradigm

Early work by Garner and colleagues established a paradigm for subsequent seductive detail studies (Garner et al., 1989, 1991). Participants studied one of two passages of text. One included only information that was directly relevant to the main topic; the other additionally included seductive details, interesting information that was not directly relevant to the main topic. The main text and seductive details were pre‐rated for both importance and interest. After studying the text, participants completed a combination of tasks, such as listing “just the really important information” they had read, rating the interestingness of the text, identifying the most interesting piece of information they had read, and matching pictures that were related to the content. Later studies included other structured and unstructured measures of recall—for example, providing a title for the passage and responding to short‐answer questions (Garner et al., 1989, 1991). Motivated by findings that learners who remember information well may not be as adept at applying that information in solving problems (Mayer et al., 1996), later studies began including problem‐solving transfer tasks (cf, Harp & Mayer, 1997). Most recent seductive detail studies include measures of both recall and transfer. This original paradigm has been adopted to study seductive details in many forms besides text and illustrations, including animations (Moreno & Mayer, 2000), photos (Sung & Mayer, 2012), video clips (Mayer et al., 2001), sounds and music (Moreno & Mayer, 2000), and details incorporated in spoken lectures (Harp & Maslich, 2005).

### 1.3 Support for the seductive detail effect

These early studies indicated that adding seductive text to a passage reduced how well participants recalled the critical content in a passage and made them more likely to remember interesting, rather than important, information (Garner et al., 1989, 1991). In some cases, the magnitude of the effect was profound. For example, when they were asked to report important information from a passage without seductive details, Garner et al. (1989) indicated that 93% of the participants reported all of the main ideas. In contrast, of participants who studied the passage with seductive details only 43% were able to list all of the main ideas. Further, they were likely to report a combination of main ideas and seductive details as important information. Not surprisingly, these studies generated a great interest in the effects of seductive details on learning. In the subsequent 30 years, many other researchers have similarly concluded that inclusion of seductive details results in participants recalling less of the critical and more of the irrelevant content (e.g., Garner et al., 1992; Lehman et al., 2007; Wade & Adams, 1990) and performing poorly on problem‐solving or transfer tasks (e.g., Harp & Mayer, 1997, 1998; Moreno & Mayer, 2000; Rey, 2014).

Several comprehensive meta‐analyses have examined the size of the seductive detail effect on both recall/retention and transfer. Rey (2012) conducted an analysis of 39 studies. Results for retention, which included 3535 participants in 34 studies, yielded a weighted mean effect size of *d* = 0.30 (99% confidence interval 0.20–0.39), a highly significant effect with a small to medium effect size. For transfer performance, covering 1634 participants in 21 studies, the weighted mean effect size was *d* = 0.48 (99% confidence interval 0.34–0.61), a highly significant value with a medium effect size. Another recent summary of 23 studies reported a median effect size of 0.86 when measuring seductive details’ effects on transfer performance (Mayer & Fiorella, 2014).

### 1.4 Inconsistent findings

Although these findings are compelling, there have been some inconsistent results. When enumerating the results, Rey (2012) found that 11 of 39 studies supported the seductive detail effect, 13 contained mixed results, and 15 did not support the effect. An earlier analysis (Thalheimer, 2004) examined results from 24 studies. Sixteen studies demonstrated that adding seductive details harmed learning, with 14 of those indicating a seductive detail effect for recall of main ideas or problem‐solving/transfer, and two showing the effect for transfer but not recall. However, of the other eight studies, seven demonstrated no seductive detail effect, and one indicated that seductive details actually helped learners recall the main ideas. A more recent meta‐analysis (Sundararajan & Adesope, 2020) examined 58 papers with 68 effect sizes involving data from 7521 participants. Here, the authors categorized results into retention‐only studies, transfer‐only studies, and studies that measured both retention and transfer. For retention, 19 results involving 2147 participants yielded a weighted mean effect size of *g* = −0.37 (95% confidence interval −0.60 to −0.13); seductive details had a significant negative effect with a small to medium effect size. For transfer performance, with six results covering 798 participants, the result was not significant and trended positive; the weighted mean effect was medium (*g* = 0.46, 95% confidence interval −0.60 to −0.13). In studies that measured both retention and transfer, there were 43 results with 4576 participants, and the weighted mean effect size was *g* = −0.41 (95% confidence interval −0.55 to −0.28); in this case, seductive details had a significant negative effect with a small‐to‐medium effect size.

Although the seductive detail effect has been found across many types of studies, the strength of the effect varies widely among the different types. In Rey's meta‐analysis (2012), seductive text studies yielded a mean weighted effect size of *d* = 0.27 for retention and *d* = 0.65 for transfer performance. Effect sizes for seductive illustrations were *d* = 0.95 for retention and *d* = 0.83 for transfer, while other types of seductive details resulted in effect sizes of *d* = 0.10 for retention and *d* = 0.18 for transfer. As noted above, effect sizes also vary for retention and transfer, particularly for seductive text. For example, one set of experiments found no performance differences on recall tests between learners exposed to high‐ or low‐interest details; however, participants exposed to high‐interest details scored lower on transfer tests (Mayer et al., 2008). Mayer (2014, p. 44) writes that he is mainly focused on transfer performance because transfer tests “can help tell us how people understand what they have learned.”

### 1.5 Methodological issues and confounds

In addition to inconsistent results, meta‐analyses have raised questions about possible confounds and methodological issues in studies of the seductive detail effect (Rey, 2012; Thalheimer, 2004; Goetz & Sadoski, 1995).

#### 1.5.1 Operational definitions

It is often difficult to find a clear, consistent operational definition of seductive details that is used across studies. The term “seductive details” was intended to apply to interesting but irrelevant details embedded in *uninteresting* text (Garner, 1992); however, some studies appear to have violated this definition by using interesting but relevant details or by embedding seductive details in material that would likely be considered inherently interesting (Goetz & Sadoski, 1995). In other words, there is not a clear distinction between the levels of importance and interest in the core text as compared to the seductive text. Further, researchers have conceptualized relevance, or importance, in different ways: while many researchers have focused on *instructional relevance* (importance in terms of the learning goals), Alexander (2019) notes that the original Garner perspective was *structural relevance* (importance in terms of how the ideas in the text are logically connected, such as by main idea and details, chronologically, step by step, etc.). Related to relevance, one study has investigated whether learners’ *perceived relevance* of seductive details influences the seductive detail effect (Eitel et al., 2019). The study showed that seductive details had a negative effect on learner performance only when learners were not told that the seductive details were irrelevant to the learning goals.

Related issues involve both inconsistent reporting of if or how content was rated for interest and relevance and inconsistent methods of rating statements and applying the terms *interesting*, *uninteresting*, *important*, and *unimportant*. Harp and Mayer (1997), for example, produced the well‐known lightning content that has been used in numerous studies of the seductive detail effect (Harp & Mayer, 1998; Kühl et al., 2019; Lehman et al., 2007; Moreno & Mayer, 2000). They defined interest as material that “readers rate … to be entertaining and interesting” and irrelevant as material that “is not related to a step in the cause‐and‐effect explanation, although it may be related to the general topic of the passage.” However, they do not include further details of the rating procedure. In other studies, more details are provided. For example, Garner et al. (1989) asked teachers to rate the statements in a text by selecting “just the important information” and “just the really interesting information”; in a later study (Garner et al., 1991), PhD students rated statements from a text as high, moderate, or low in both interest and importance (but further criteria were not specified).

Importantly, some researchers have suggested systematic rating processes for evaluating the interests and importance of content. For example, in one procedure, raters read a text passage and then separately read each sentence from the passage (Wade & Adams, 1990). They were asked to identify one‐fourth of the sentences as “not at all interesting” using a four‐point, Likert‐type scale (1 = not at all interesting, 4 = very interesting). They repeated the process to rate one‐fourth of the sentences as 2s, and so on with 3s and 4s. The process was then repeated for importance. Mean scores were calculated across raters for each sentence for both interest and importance, with scores below the median being labeled high and above the median being labeled low. That yielded four sentence categories: high importance/high interest, high importance/low interest, low importance/high interest, and low importance/low interest. In another procedure, raters read a text passage and were asked to rate their interest in each of the sentences using the same four‐point scale noted above (Lehman et al., 2007); the raters repeated the process to rate each sentence for its importance to the overall meaning of the passage. Means were calculated, and a median split was used to separate statements into high and low groups based on both importance and interest. High interest/low importance statements were classified as seductive details; the remaining statements were considered to be base text. Thus, standard processes are available for rating interest and importance.

In summary, all of this points to the necessity of studies reporting the rating process, of using standard definitions of terms such as “interest” and “importance,” and of rating both the core content and the seductive details according to those definitions.

#### 1.5.2 Passage length and reading level

One of the issues criticized in the early seductive detail studies was the fact that text passages containing seductive details were significantly longer than the passages that did not contain seductive details. For example, in the Garner et al.’s (1989) study, the passage containing seductive detail sentences was nearly 40% longer than the base passage (Goetz & Sadoski, 1995). This creates the possibility that learners failed to remember the main ideas in the seductive detail passages simply because there was more text to process. Since they had received no cues as to what was important, the longer seductive detail passages potentially obscured or minimized the potency of the main ideas.

Researchers have addressed the issue of mismatched passage lengths in two main ways. One study incorporated both high‐interest and low‐interest details of approximately the same number of words so that passage lengths would be fairly equal (Mayer et al., 2008). In the same study, the researchers determined that “highly interesting details may be inherently longer”; to compensate for this, participants were allowed to study the lesson for as much time as needed. Most seductive detail studies since then have used these approaches.

A related issue is reading level. While many seductive detail studies report the reading level of the overall passages/core content, our review of the literature yielded no studies that separately reported the reading level of the core content and the seductive details. Further, reading levels of the high‐ and low‐interest seductive detail statements have not typically been reported or compared. Given Mayer et al.’s (2008) suggestion that high‐interest details tend to be longer, coupled with the fact that sentence length is one determinant of reading level, it is possible that highly interesting seductive details are also more difficult to read. This makes it difficult to determine whether any seductive detail effect is driven by interest, reading difficulty, or a combination of the two factors.

#### 1.5.3 Learning objectives

Most studies in the seductive detail literature do not include learning objectives, even though students are accustomed to materials—such as textbooks—in which objectives are provided. Yet, in unstructured recall tests, participants are typically asked to recall only the really important information (Garner et al., 1989). It could be that learners did not report some of the important information they remembered because they did not recognize it as being important. Instructional objectives establish which instructional material is relevant to the learning task and which material can be considered extraneous details (Rey, 2012). One study found that when learning objectives were provided, performance on material related to the objectives improved by more than 45% over situations in which learning objectives were not used (Rothkopf & Billington, 1979). It seems reasonable to expect materials to guide learners in distinguishing which information is important enough to warrant their attention (Goetz & Sadoski, 1995). One study incorporated learning objectives but did not manipulate or test them (Park et al., 2011). Another study that provided learning objectives indicated that adding the objectives did not reduce the seductive details effect, but did help learners to score higher on both tests of their recall of main ideas and on tests of transfer skills (Harp & Mayer, 1998).

#### 1.5.4 Prior knowledge, working memory, and cognitive load

Learners who have a high level of prior knowledge about a subject area may be less susceptible to the seductive detail effect because they already know which information is important and which is irrelevant. However, many seductive detail effect studies did not directly test participants’ prior knowledge of the lesson content but used only self‐assessment as a gauge (Harp & Mayer, 1997). An exception was Garner et al. (1991) who found that participants with higher levels of domain knowledge performed better on recall measures. In most studies, prior knowledge did not appear to be used as a covariate in statistical analyses (Rey, 2012).

Learners who are high in working memory capacity may also be less susceptible to the seductive detail effect and, in fact, have been shown to perform better when seductive details are included in a lesson (Sanchez & Wiley, 2006). One study found no general differences in outcomes between learners in a seductive detail study conducted in a classroom, but did indicate that learners who had more prior knowledge and were higher in working memory capacity appeared to benefit from the seductive details (Maloy et al., 2019). Another contributor to inconsistent results may be cognitive load imposed by the content; participants in a low‐load condition who were exposed to seductive details performed better than those who were not (Park et al., 2011). Related to this topic, a recent study reported effects of perceptual load on the seductive detail effect: while no seductive detail effect was evident in high perceptual load conditions, learners in low perceptual load conditions who were exposed to seductive details did not perform as well as those not exposed to seductive details (Wang et al., 2021).

#### 1.5.5 Arousal/valence

Several recent studies have focused on potential emotional effects related to seductive details. It is possible that the valence of the emotion—negative or positive—in the details or the learner's state of arousal could influence the seductive detail effect, although this is not clear. A recent study found that emotional valence neither hindered or fostered the seductive detail effect (Kühl et al., 2019). One study demonstrated that induced negative emotions in learners had a facilitating influence on learning outcomes, while induced positive emotions had a suppressing influence (Knörzer et al., 2016). Another study showed that a learner's level of arousal can moderate the seductive detail effect (Schneider et al., 2019). It is possible that a confound between emotional interest level (arousal) and emotional valence could make this difficult to interpret.

### 1.6 The current study

The current study was modeled after prior studies (e.g., Garner et al., 1989; Harp & Mayer, 1998; Mayer et al., 2008; Park et al., 2015) in order to determine whether a seductive detail effect would be manifest if some of the confounds and methodological issues were addressed. Specifically, learning objectives were incorporated to test whether the availability of objectives reduces the seductive detail effect, confounds such as word count were eliminated, a specific definition of “seductive” was used, a test of prior knowledge was incorporated, clear requirements and a well‐defined process were established for rating both the core text and the extraneous details based on importance and interest levels, and all text was matched based on reading level.

This study was based on a multimedia lesson about plate tectonics that contained a core set of content and either no extraneous details, low‐interest details, or high‐interest (seductive) details. Participants were tested on their recall of the core content and of the details and also took a transfer skills test.

The hypotheses for the study were as follows:

- H1: Participants exposed to learning objectives will score higher in core content recall and in transfer skills performance.
- H2: Participants exposed to high‐interest details will score lower in core content recall and in transfer skills performance than those in the no‐details or low‐interest details condition.
- H3: Participants exposed to high‐interest details, but not exposed to learning objectives, will show the lowest transfer skills performance.
- H4: Participants exposed to high‐interest details will report higher levels of cognitive load than those in the low‐ or no‐details conditions.

## 2 MATERIALS AND METHODS

### 2.1 Design

The study utilized a 3 × 2 design with detail type (none, low‐interest, or high‐interest) and learning objectives (exposed to or not) as between‐subject factors. Detail types and learning objectives were combined in all possible ways to create six different conditions, and 22 participants were randomly assigned to each of the six conditions.

### 2.2 Participants

A power analysis (Ellis, P. D., 2012) informed the sample size required to achieve a medium effect size. Participants were 132 undergraduate students (35 women) recruited from the undergraduate psychology subject pool. All were native English speakers between the ages of 18 and 30 (*M*_age _= 19.9, *SD *= 0.5). Twenty‐two participants had previously taken a class in geophysics, geology, or geological engineering, and one participant majored in one of these areas.

### 2.3 Materials

#### 2.3.1 Lesson content

The lesson consisted of ten screens, eight presenting text and static images and two providing instructions and references. Each content screen was related to at least one of the learning objectives shown below; all objectives related to the core content and not to the extraneous details.

- Define terms related to plate tectonics, such as mantle, crust, subduction, and supercontinent.
- Define the plate tectonics theory and explain what causes plates to move.
- Identify the three types of plate boundaries, and describe the plate movement at each boundary type.
- Name three areas on Earth that are changing due to plate movement and indicate what type of geophysical activity might be expected to occur at each location.

**FIGURE 2:**
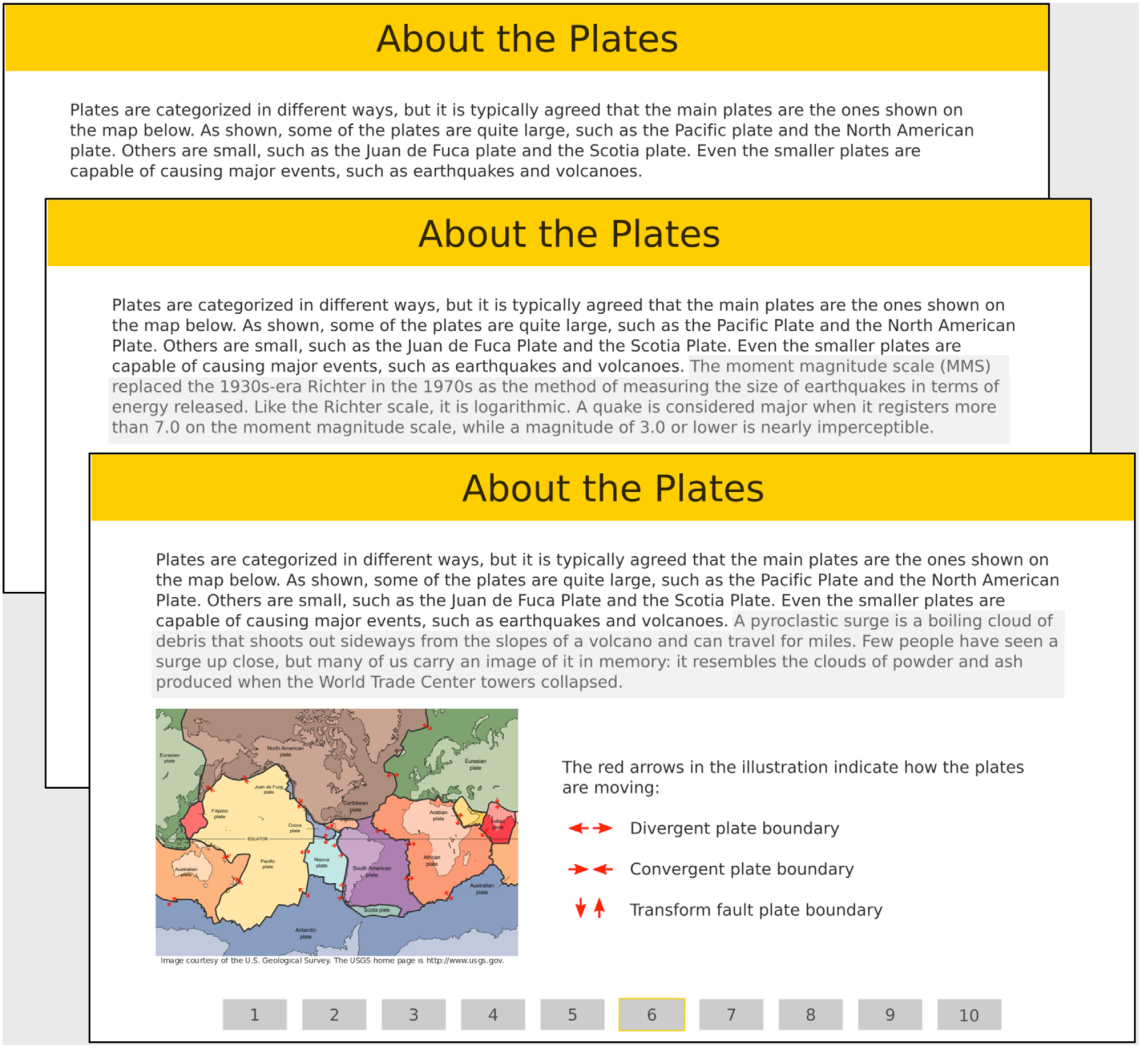
Lesson content for screen 6: with no extraneous details (top), with the low‐interest detail (center, the shaded area at the end of the first paragraph), and with the high‐interest detail (bottom, the shaded area at the end of the first paragraph)

Three versions of the lesson were created, one containing no extraneous details (Figure 2, top), one containing low‐interest details (Figure 2, center), and one containing high‐interest details (Figure 2, bottom). All details were in the form of text; the illustrations used in the lesson were directly related to the core content and were not considered extraneous. Extraneous details were incorporated at appropriate places, to blend in well with the core content, and were not flagged or highlighted in any way. Low‐ and high‐interest details were placed in the same position on their respective pages if they fit with the flow of the content or, if not, as close to the same position as possible.

The eight content screens contained a total of 987 words of core content. The low‐interest detail and high‐interest detail versions included an additional 458 words and 464 words, respectively.

#### 2.3.2 Extraneous details

To ensure the details were appropriately rated as high‐interest and low‐importance as per the categories specified by Wade and Adams (1990), a set of potential details was written for each page in the lesson, with an eye toward where they could be incorporated on the page. The adapted versions of Wade and Adams’ four categories were high importance/medium interest (main ideas), low importance/medium interest (supporting details), low importance/high interest (high‐interest seductive details), and low importance/low interest (low‐interest extraneous details).

To aid in the selection of details that were of lower importance than the core content and identification of well‐differentiated low‐ and high‐interest details, a pilot study—modeled after Lehman et al. (2007)—was conducted online through SurveyMonkey. Survey participants were United States citizens between the ages of 18 and 30, with at least a high school diploma. Fifty‐five people (24 women) completed the study and correctly answered the trap questions.

Participants first read the objectives and plate tectonics lesson with no extraneous details. Next, participants rated the interest level and importance of all of the core content text and a set of extraneous details. Mean importance and interest scores were calculated for all detail statements, and a median split was used to distinguish the low/high importance and interest statements. Mean scores were also calculated for the core content statements. The high‐interest details selected for use in the lesson were the statements that ranked high in interest and low in importance; in addition, they were required to be higher in interest and lower in importance than the mean scores for the core text. The word counts and reading levels of the low‐ and high‐interest statements were also closely matched. The mean interest and importance ratings for the core content and the 16 selected details are shown in Table 1.

**TABLE 1:** Mean interest level, importance rating, and reading level for the core content and 16 selected details used in the study, plus word counts for the low‐ and high‐interest details

Text	Interest	Importance	Word Count	Reading Level
Core content	4.69	5.50		8.85
Low‐interest details	4.11	4.15	57.25	10.19
High‐interest details	5.37	4.12	58.00	10.16

#### 2.3.3 Stroop task

Working memory was assessed using the numerical Stroop task from the Psychology Experiment Building Language (PEBL) (Mueller & Piper, 2014). In this task, participants view numbers on the screen and indicate the total number of characters they see (Hernández et al., 2010). On congruent trials, the number of characters is the same as the presented character (e.g., “333” requires a response of “3”); on incongruent trials, the number of characters is different (e.g., “222” requires a response of “3”). The Stroop interference score (Kane & Engle, 2003; MacLeod, 1991), calculated as incongruent response time (RT) minus congruent RT, served as the measure of working memory capacity, with a higher interference score indicating a lower level of working memory capacity.

#### 2.3.4 Pretest

The pretest comprised two multiple‐choice and two short‐answer questions related to plate tectonics.

**FIGURE 3:**
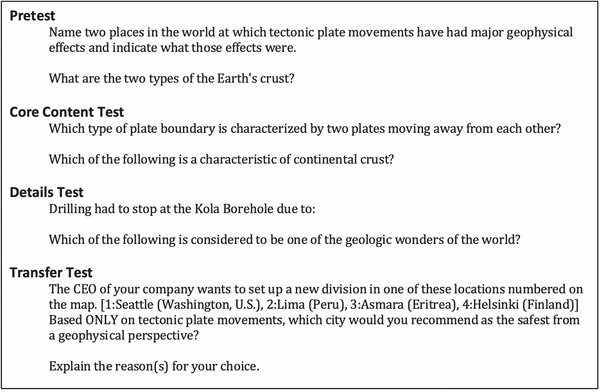
Examples of questions used in the current study's tests

All tests were created, assembled, and displayed using SurveyMonkey. Figure 3 provides sample questions for each of the tests.

##### 2.3.4.1 Core content test

The core content test included 10 multiple‐choice questions. The questions were all related to the core lesson content and not to the extraneous details. The order of the questions was randomized for each user.

##### 2.3.4.2 Details test

The details test included 16 questions, eight covering low‐interest and eight covering high‐interest details. All detail questions were delivered to all participants, providing a means of checking how well participants could guess the answers to questions about the details they did not see. For example, participants who had viewed the high‐interest details in the lesson were presented with questions on both the high‐ and low‐interest details; in addition, participants who saw no extraneous details also took the details test to provide a baseline guessing rate.

##### 2.3.4.3 Transfer test

The transfer test contained one multiple‐choice question and three short‐answer questions, one of which had two parts. The questions all related to the core content, and not to the extraneous details.

#### 2.3.5 Participants’ perceived level of cognitive load

Following the lesson, participants used a seven‐point scale (extremely low to extremely high) to rate the mental effort they thought they had to expend while studying the lesson. Participants responded to a single item: “While studying the lesson, my mental effort was…”

Table 2 lists all of the study's dependent measures.

**TABLE 2:** Dependent measures collected in the experiment

Measure	Values
Stroop task interference time/working memory	Incongruent time minus congruent time in milliseconds
Pretest score	Score range: 0–5 points
Study time on lesson content screens	Time in minutes/seconds
Cognitive load rating	Range: 1–7
Core content test score	Score range: 0–10 points
Details test performance, high‐interest details	Score range: 0–8 points
Details test performance, low‐interest details	Score range: 0–8 points
Transfer test score	Score range: 0–5 points

### 2.4 Procedure

After signing the consent form, participants completed the demographics form, Stroop task, and pretest. Next, participants in the objectives condition received a printed list of learning objectives to reference during the lesson. The experimenter read through the list of objectives with each participant and explained that the list contained the information they were expected to learn and that the information may appear on the tests. Participants were not permitted to take notes during the lesson.

The lesson was presented via the E‐Prime® software. Participants could move to the next screen by pressing the spacebar, but could not return to previous screens. The software tracked study time, which was not limited.

After the lesson, the experimenter collected the objectives list (if applicable), and the participant completed either the core content test or the transfer skills test (the order was counterbalanced); participants were given 10 min to complete either test. Next, participants took the details test, which they had 16 min to complete. Finally, participants took either the core content or transfer test, whichever one they had not already taken. The entire experiment took less than 60 min to complete.

## 3 RESULTS

### 3.1 Analysis

Test scores and cognitive load measures were analyzed in 3 × 2 analysis of covariance (ANCOVA) with detail type (none, low‐interest, or high‐interest) and objectives (exposed to or not) as between‐subject factors. To control for each participant's level of working memory, Stroop interference scores were included as a covariate. Statistical tests were conducted both with and without the covariate; since it had an effect in some cases, ANCOVA results are reported and effects of working memory are noted. For all post hoc *t*‐tests, reported *p*‐values reflect Bonferroni adjustments.

Data (Tislar & Steelman, 2020) were excluded from 22 participants who had either majored or taken college‐level courses in geophysics, geology, or geological engineering, due to higher mean pretest scores (3.82 vs. 3.05) and near ceiling performance on the core content test. This resulted in 54 participants in the no‐objectives condition (16 saw high‐interest details, 18 saw low‐interest details, and 20 saw no details) and 58 participants in the objectives condition (18 saw high‐interest details, 22 saw low‐interest details, and 18 saw no details).

### 3.2 Working memory

Data from the Stroop task were used to calculate a measure of working memory. The mean interference score was 74.51 ms (*SD *= 33.64). An ANOVA indicated no significant interference score differences among groups based on either detail type, *F*(2, 126) = 0.17, *p *= .84, *η_p_* ^2 ^= 0.003, or objectives, *F*(1, 126) = 0.25, *p *= .62, *η_p_* ^2 ^= 0.002. There were also no differences based on an interaction between the two factors, *F*(2, 126) = 0.41, *p *= .67, *η_p_* ^2 ^= 0.01. Although the condition groups are well matched for working memory, working memory is included as a covariate in subsequent analyses as it accounts for some of the score variances within groups.

### 3.3 Pretest for prior knowledge

The mean pretest score was 3.05 (*SD *= 1.07). No pretest question was answered correctly by every participant, and performance was above chance on both of the multiple‐choice questions. An ANCOVA indicated no difference in pretest scores among groups based on either detail type, *F*(2, 103) = 0.69, *p *= .5, *η_p_* ^2 ^= 0.01, or objectives *F*(1, 103) = 0.52, *p *= .47, *η_p_* ^2 ^= 0.01, controlling for working memory. The effect of working memory was not significant, *F*(1, 103) = 0.04, *p *= .85, *η_p_* ^2 ^< 0.001. In addition, there were no differences based on an interaction between detail type and objectives, *F*(2, 103) = 0.39, *p *= .68, *η_p_* ^2 ^= 0.01.

### 3.4 Study time

An ANCOVA indicated a significant main effect of detail type on study time *F*(2, 103) = 3.1, *p *= .05, *η_p_* ^2 ^= 0.06, with participants spending significantly more time studying in the low‐ or high‐ interest detail conditions (*p *= .04 in both cases) than in the no‐detail condition. However, a follow‐up analysis of the effect of detail type on reading rate (see Table 3) indicated that participants did not spend more time than would be expected based only on the additional number of words included in those conditions, *F*(2, 103) = 1.52, *p *= .22, *η_p_* ^2 ^= 0.03.

**TABLE 3:** Means and standard errors for reading rates per page, in words per second

Condition	Mean	Standard error
Viewed low‐interest details (*N* = 38)	2.8	0.85
Viewed high‐interest details (*N* = 34)	2.9	1.09
Viewed no details (*N* = 38)	2.51	1.05

There was no significant effect of objectives on study time, *F*(1, 103) = 0.28, *p *= .6, *η_p_* ^2 ^= 0.003, and no interaction between the two factors, *F*(2, 103) = 1.01, *p *= .37, *η_p_* ^2 ^= 0.02. The effect of working memory on study time was significant, *F*(1, 103) = 5.00, *p *= .03, *η_p_* ^2 ^= 0.05, with lower working memory capacity associated with longer study times.

Notably, across all three conditions, there was no significant relationship between study time and scores on any of the three tests (core content: *r *= 0.03, *n *= 110, *p *= .73; details: *r *= 0.06, *n *= 110, *p *= .51; transfer skills: *r *= 0.14, *n *= 110, *p *= .14).

### 3.5 Cognitive load/mental effort

On average, participants reported their level of mental effort was 3.95 (*SD *= 1.03) while completing the lesson. An ANCOVA indicated no significant main effects of either detail type, *F*(2, 103) = 0.13, *p *= .88, *η_p_* ^2 ^= 0.003, or objectives, *F*(1, 103) = 0.15, *p *= .7, *η_p_* ^2 ^= 0.001, nor a significant interaction between the two factors, *F*(2, 103) = 1.62, *p *= .20, *η_p_* ^2 ^= 0.03. The effect of working memory on mental effort was significant, *F*(1, 103) = 3.94, *p *= .05, *η_p_* ^2 ^= 0.04; lower levels of working memory capacity were associated with higher levels of mental effort.

Although a higher reported level of mental effort was associated with a higher score on the transfer skills test, *r *= 0.20, *n *= 110, *p *= .04, there was no significant relationship between mental effort and the core content test score, *r *= 0.03, *n *= 110, *p *= .78, or the detail test score, *r *= –0.17, *n *= 110, *p *= .08.

### 3.6 Core content test

**FIGURE 4:**
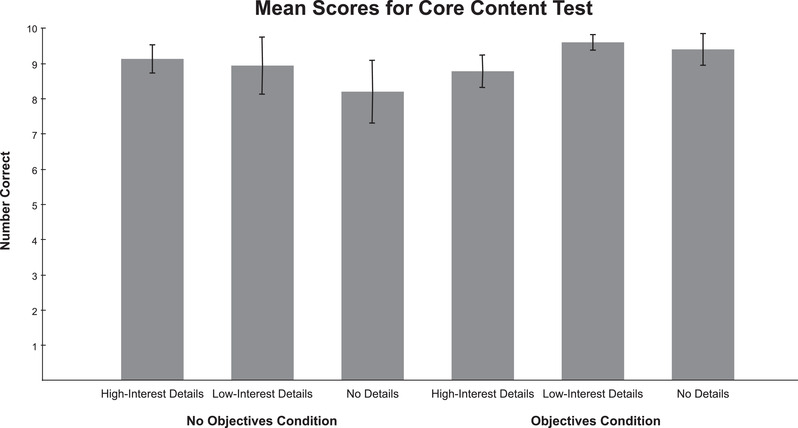
Participant scores on the core content test. Error bars represent 95% confidence intervals

Participants across all conditions scored extremely high on the core content test, with a mean overall score of 9.01 (*SD *= 1.37). No question was answered correctly by every participant, and performance was above chance on all questions. Figure 4 graphs the mean scores by condition.

An ANCOVA of the scores indicated no significant main effect of detail type, *F*(2, 103) = 1.19, *p *= .31, *η_p_* ^2 ^= 0.02, controlling for working memory. There was an effect of objectives, *F*(1, 103) = 4.8, *p *= .03, *η_p_* ^2 ^= 0.05, with higher scores attained when objectives were available. There was also a significant interaction between detail type and objectives, *F*(1, 103) = 3.55, *p *= .03, *η_p_* ^2 ^= 0.07.

To identify the source of the interaction, additional ANCOVAs were run separately for the no‐objectives and the objectives conditions. When objectives were not available there was no significant effect of detail type, *F*(2, 50) = 1.78, *p *= .18, *η_p_* ^2 ^= 0.07. When objectives were available, there was a significant effect of detail type, *F*(2, 52) = 4.82, *p *= .01, *η_p_* ^2 ^= 0.16. Post hoc *t*‐tests revealed that scores were higher in the low‐interest details condition (*M *= 9.6, *SD *= 0.50) than in the high‐interest details condition (*M *= 8.78, *SD *= 1.0); *t*(36) = −3.24, *p *< .01, *d *= 1.05; this is consistent with the seductive detail effect. (Here and in the following paragraphs, *t‐*test effect sizes are reported as Cohen's *d*.) There was also a significant difference between the no‐details (*M *= 9.44, *SD *= 0.98) and the high‐interest detail conditions (*M *= 8.78, *SD *= 1.00); *t*(34) = −2.01, *p *= .05, *d *= 0.67. There was no significant difference between the no‐details and low‐interest conditions.

The effect of working memory on the core content test score was not significant, *F*(1, 103) = 1.11, *p *= .3, *η_p_* ^2 ^= 0.01.

### 3.7 Transfer skills test

None of the transfer skills questions were answered correctly by every participant, and performance was above chance on the multiple‐choice question.

**FIGURE 5:**
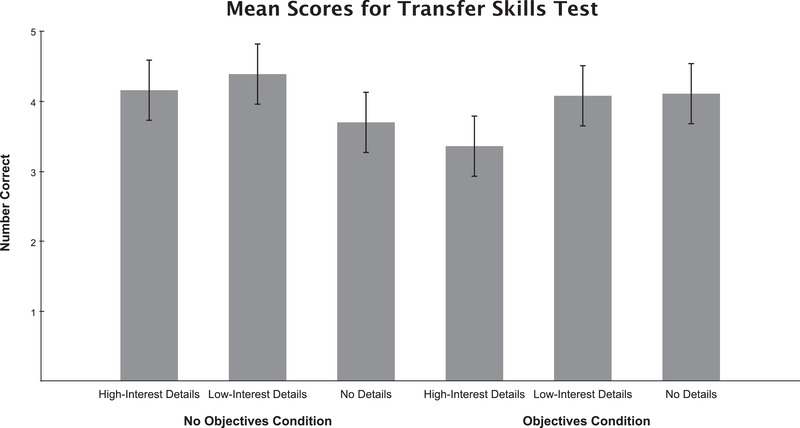
Participant scores on the transfer skills test, which had a possible score of five points. Error bars represent 95% confidence intervals

Figure 5 illustrates the transfer skills test scores for each condition. An ANCOVA revealed no main effects of either detail type, *F*(2, 103) = 2.11, *p *= .13, *η_p_* ^2 ^= 0.04, or objectives *F*(1, 103) = 1.17, *p *= .28, *η_p_* ^2 ^= 0.01. The effect of working memory was not significant, *F*(1, 103) = 0.49, *p *= .49, *η_p_* ^2 ^= 0.01. There was, however, an interaction between detail type and objectives *F*(2, 103) = 3.39, *p *= .04, *η_p_* ^2 ^= 0.06.

To identify the source of the interaction, additional ANCOVAs were run separately for the no‐objectives and the objectives conditions. When objectives were available, there was a nonsignificant effect of details *F*(2, 52) = 2.80, *p *= .07, *η_p_* ^2 ^= 0.1; however, post hoc *t*‐tests indicated a trend toward a seductive detail effect with lower scores in the high‐interest condition (*M *= 3.36, *SD *= 1.27) than in the low‐interest condition (*M *= 4.08, *SD *= 1.12); *t*(36) = −1.84, *p *= .07, *d *= 0.6. In addition, there was a significant difference between the no‐details scores (*M *= 4.11, *SD *= 0.76) and the high‐interest detail scores (*M *= 3.36, *SD *= 1.27); *t*(34) = −2.15, *p *= .04, *d *= 0.72. There was not a significant difference between the no‐details and low‐interest detail scores.

In the no‐objectives condition, there was again a nonsignificant effect of detail type, *F*(2, 50) = 2.80, *p *= .07, *η_p_* ^2 ^= 0.1. In contrast to the objectives condition, there was no evidence for a seductive detail effect, with no difference in transfer scores between the low‐ and high‐interest detail conditions, *t*(32) = –0.9, *p *= .38, or between the no‐details and high‐interest detail conditions, *t*(34) = 1.28, *p *= .21. However, a post hoc *t*‐test revealed a significant difference between the no‐details and the low‐interest detail conditions, *t*(36) = 2.18, *p *= .04, *d *= 0.71. Notably, the direction of this effect is opposite of what one would expect from a word‐count effect (no‐details scores: *M *= 3.7, *SD *= 1.2; low‐interest detail scores: *M *= 4.39, *SD *= 0.63).

### 3.8 Details test

None of the high‐interest detail or low‐interest details questions were answered correctly by all participants. Performance was above chance on all questions.

#### 3.8.1 High‐interest detail questions

An ANCOVA for the high‐interest details score indicated a main effect of detail type, *F*(2, 103) = 13.68, *p *< .001, *η_p_* ^2 ^= 0.21. Consistent with expectations, those in the high‐interest group scored significantly higher than those in the no‐details condition *t*(70) = 5.31, *p *< .001; however, they did not score significantly higher than those in the low‐interest detail group. Scores in both the low‐interest and high‐interest detail conditions were higher than in the no‐detail condition (both *p *< .001).

There was no significant main effect of objectives, *F*(1, 103) = 0.25, *p *= .62, *η_p_* ^2 ^= 0 or interaction between the objectives and detail type, *F*(2, 103) = 2.07, *p *= .13, *η_p_* ^2 ^= 0.04. The effect of working memory was not significant, *F*(1, 103) = 2.55, *p *= .11, *η_p_* ^2 ^= 0.02.

#### 3.8.2 Low‐interest detail questions

An ANCOVA run on the low‐interest detail scores indicated a main effect of detail type, *F*(2, 103) = 8.2, *p *< .001, *η_p_* ^2 ^= 0.14. Consistent with expectations, participants who saw the low‐interest details scored higher on the low‐interest detail questions than those in either the high‐interest detail (*p *= .04) or no‐detail condition (*p *< .001). There was no main effect of objectives in the low‐interest detail questions, *F*(1, 103) = 0.38, *p *= .54, *η_p_* ^2 ^= 0.00 and no interaction between details and objectives, *F*(2, 103) = 0.85, *p *= .43, *η_p_* ^2 ^= 0.02. The effect of working memory was not significant, *F*(1, 103) = 1.4, *p *= .25, *η_p_* ^2 ^= 0.01.

Table 4 lists mean scores for each condition, and Table 5 provides descriptive statistics for all study measures.

**TABLE 4:** Comparison of means and standard deviations for scores on details test questions according to the type of detail content that was viewed, collapsed across objectives

	Low‐interest details score		High‐interest details score	
Condition	Mean	SD	Mean	SD
Viewed low‐interest details (*N *= 40)	5.53	1.43	4.82	1.41
Viewed high‐interest details (*N *= 34)	4.71	1.47	5.06	1.01
Viewed no details (*N *= 38)	4.26	1.29	3.68	1.16

**TABLE 5:** Means and standard deviations for study measures

Measure	Mean	Standard deviation
Pretest	3.05	1.07
Study time (minutes)	8.86	3.26
Core content test	9.01	1.37
Details test	9.34	2.18
Transfer test	3.96	1.04
Working memory (milliseconds)	74.51	33.64
Cognitive load	3.95	1.03

## 4 DISCUSSION

This study was designed to investigate whether the seductive detail effect documented in prior studies would emerge when a specific set of confounds and methodological issues was addressed. When confounds noted in the literature—word count and reading level—were controlled for, extraneous details were carefully selected on the basis of both interest and importance, prior knowledge was established using a test, and learning objectives were incorporated to help guide users about which information was important, the study did not reveal a consistent detrimental effect of high‐interest details on core content recall and transfer skills scores; therefore, Hypothesis 2 was not supported. However, a seductive detail effect was observed in some very specific scenarios. For the core content test, an interaction between detail level and objectives availability emerged, with a significant seductive detail effect manifesting only when objectives were provided. A similar interaction occurred in transfer skills test scores, and there was a trend toward a seductive detail effect, again, only when objectives were provided. For transfer scores in the no‐objectives condition, there was no evidence for a seductive detail effect, with no significant difference in scores between the low‐ and high‐interest detail conditions or between the no‐details and high‐interest detail conditions. There was, however, a significant difference between the no‐details and the low‐interest detail scores, with higher scores in the low‐interest detail condition.

The patterns of results described above are not consistent with Hypothesis 1: we predicted that participants exposed to learning objectives would perform better than participants who were not exposed, and this was not the case. Hypothesis 3 predicted that, in the no‐objectives condition, participants exposed to high‐interest details would score lower than those in the other two conditions; there was no support for this hypothesis.

Although the influence of learning objectives on the seductive detail effect has not been widely studied, two prior studies found that providing objectives greatly increased learner performance (Harp & Mayer, 1998; Rothkopf & Billington, 1979). A study by McCrudden (2019) incorporated pre‐reading questions, similar to the current study's conceptualization of learning objectives that focused on the main ideas of the text (which were more relevant to the task but less interesting). Consistent with the other studies noted above, these pre‐reading questions improved recall of the main ideas even when seductive details (which were less relevant to the task, but more interesting) were present. In contrast, the current study yielded a significant interaction of objectives availability and detail type on both the core test and transfer skills test results: participants exposed to learning objectives scored higher in both the no‐details and low‐interest details conditions, but not in the high‐interest details condition. Why did objectives seem to enhance the seductive detail effect in the high‐interest detail condition? One possibility considered is that maintaining the objectives in working memory while studying the material added to learners’ cognitive load, which we expected to be highest in the high‐interest detail condition. However, the cognitive load ratings gathered during this study provide no support for that supposition, refuting Hypothesis 4.

Since these results in the high‐interest detail condition are contrary to expectations, further investigation is needed to establish why the objectives were ineffective (or possibly detrimental) in this condition and why results differed from those of the McCrudden's study.

The current study utilized a set of seductive details that was selected based on a systematic evaluation. The process, which was based on previous studies (Wade & Adams, 1990; Lehman et al., 2007), involved rating the interest‐level of both the ideas in the core text and the low‐ and high‐interest details. The relevance of both the details and the core text to the information specified by the learning objectives was also rated, such that all text used in the lesson could be ranked on the basis of interest level and importance. Could it be that the high‐interest seductive details used in this study were not sufficiently interesting to elicit the seductive detail effect across both of the objectives conditions? This raises an interesting problem for further research: just how interesting does the seductive information need to be, and how can interest be properly measured so as to allow comparisons among studies and provide guidance to practitioners?

Although high‐interest seductive details were always rated as significantly more interesting than low‐interest details and the core content in this study, the extant literature does not provide standard definitions or guidance as to how interesting a detail must be to qualify as a seductive detail. Additional research should examine other dimensions that could be used in developing and rating details, such as a scale based on Schraw and Lehman's (2001) personal versus situational interest (a desire to understand a topic that persists over time vs. interest that is spontaneous and context‐specific). Also, some details may seem more or less interesting when they are read in context than when they are read as stand‐alone statements in ratings studies; it may be worthwhile to develop a method of rating the details in context.

Another potential method of rating details is according to the type of interest they evoke. There are two main types of interest involved in reading text, according to Kintsch (1980): cognitive interest and emotional interest. Cognitive interest is engaged by content that helps the readers understand the material, such as explanatory summaries, or that helps them to make connections among the pieces of information they have been given. Emotional interest can increase readers’ emotional arousal and help them to focus more on the content, which ideally would lead to increased learning. Generating text that evokes emotional interest is often accomplished by including extraneous information about topics such as death, power, money, and sex (Kintsch, 1980). Although Kintsch thought that material should be balanced between emotional and cognitive interest, it can be difficult to come up with emotionally interesting information about many domains, including plate tectonics. Some of the details in the current study are related to death, while others are related to interesting places around the world and earthquake and volcano sites in the United States, which could potentially increase emotional arousal. Overall, although, the details in the current study may be more cognitively than emotionally interesting.

As previously noted, the seductive detail effect may be influence by emotional interest level and valence. Given the fact that the details in the current study with an emotional component are related to death and destruction, they likely evoke negative emotions, which could obscure any seductive detail effect. This suggests the importance of matching the emotional valence level of the high‐ and low‐interest details.

In the current study, the low‐interest details are not technically seductive details according to the standard definition (Garner et al., 1991) because each one was rated as numerically less interesting than the core text. The low‐interest details were not intended to provide supporting material for the core content; however, they bring to mind Ellis’ concept of “catalytic” content (Ellis, J., 2012). He contends there is a category of content that is added to text passages not because it directly relates to the learning objectives or is of particular interest to learners, but because it “introduces, supports, contextualizes, exemplifies, or reinforces that primary content which is relevant and essential in terms of addressing or achieving the learning outcomes.” It could be that some of the extraneous details are inadvertently catalytic and end up being beneficial to learning. Maybe that is one reason for several studies finding positive effects of seductive details under certain conditions (Garner et al., 1991; Ketzer‐Nöltge et al., 2019; Lehmann et al., 2019). If indeed catalytic content plays a role here, then it may be another confound that has not been addressed in prior studies. This would add to the difficulty of writing content for the control condition that matches the seductive detail condition in word count and reading level, but is less interesting and noncatalytic.

The current study demonstrated the difficulty of writing details that were seductive under any condition. Despite the fact that details were carefully written with both emotional and cognitive interest and were pre‐tested for both interest level and relevance, the observed effects were much smaller than those reported in prior meta‐analyses. It is unclear whether the current results are driven by some aspect of the content or of the details, but effective guidelines for the use of seductive details will need to take both factors into account.

The inconsistent results in the current study align with several other studies that have cast doubt on the generalizability of the seductive detail effect. As noted earlier, of 39 studies examined in a meta‐analysis, 11 supported the seductive detail effect, 13 contained mixed results, and 15 did not support the effect (Rey, 2012). More recently, a special issue of Applied Cognitive Psychology (Eitel & Kühl, 2019a) contained 11 papers related to the seductive detail effect: Five studies supported the effect, two found a beneficial effect, two did not support the effect, and two did not directly test the effect. Notably one of the studies that failed to observe the effect (Kühl et al., 2019) utilized the well‐traveled lightning content which had yielded seductive detail effects in prior studies (Harp & Mayer, 1997, 1998). In light of the inconsistent results in the special issue, Eitel and Kühl (2019b) suggest that “there is no unconditional negative effect of seductive details; but rather, that the effect is bound to specific conditions.”

## 5 CONCLUSIONS

As evidenced by the current study, and by the recent special issue of Applied Cognitive Psychology (Eitel & Kühl, 2019a), many studies have found null or beneficial effects of seductive details. Although Mayer still asserts that “adding interesting but irrelevant material to a lesson hurts learning” (Mayer, 2019, p. 141), we hold that this admonition needs to be qualified. The current study, recent publications in the special issue, and the meta‐analyses all highlight the fact that the seductive detail effect is mediated by a variety of factors. Unfortunately, examining some of these factors seems to draw the seductive detail research more into manufactured methods and materials that are unrelated to how and what students normally read. As Alexander writes in her review of the special issue, the seductive detail research needs to have “more direct relevance to typical learners’ reading of typical texts under typical conditions” (Alexander, 2019, p. 147). We add that the research should either demonstrate methods or results in practical guidelines that would enable typical instructors to make informed decisions about using seductive details. While it is true that researchers must often do atypical manipulations in order to elicit effects in the lab, in this case such manipulations seem to be producing unworkable heuristics—such as a ban on using seductive details or an expectation that instructors can somehow control everything that affects their materials. For example, it is not typical for instructors to have each sentence they use in their materials evaluated for interestingness and relevance, to assess each statement's reading level and word count, or to point out exactly which information is not entirely relevant to the learning goals.

Until more specific guidelines can be developed, educators’ time may be better spent designing learning materials that take advantage of other well‐tested instructional design principles such as the modality principle (Low & Sweller, 2014) and the signaling principle (Mayer & Fiorella, 2014) than combing through their materials to excise potential seductive details. We echo the recent suggestion of Alexander (2019) that it would be far more beneficial for educators to simply write learning material that is cohesive, concise, and engaging.

The current study highlights concerns about aspects of the seductive detail effect, including the definitions related to seductive details and potential mediating factors such as the availability of learning objectives. Our reason for conducting seductive detail studies was that we could provide educators with clear, workable guidelines for how/when/if seductive details should be handled. However, more research is required in these areas before general guidelines can be provided. Given the fact that the effect does not seem to be as straightforward as prior research has implied, that the estimated size of the seductive detail effect may be inflated due to publication bias (Kühl et al., 2019), and that the little research that has been done has not demonstrated the seductive detail effect outside of the lab (Muller et al., 2008; Maloy et al., 2019), perhaps educators should not be overly worried about including interesting, but irrelevant, information in their instructional materials.

### PEER REVIEW

The peer review history for this article is available at https://publons.com/publon/10.1002/brb3.2322.

## CONFLICT OF INTEREST

The authors declare no conflict of interest.

## Data Availability

The data that support the findings of this study are openly available in Digital Commons @ Michigan Technological University at https://digitalcommons.mtu.edu/data‐files/2.
